# Host Genetic Variants and Gene Expression Patterns Associated with Epstein-Barr Virus Copy Number in Lymphoblastoid Cell Lines

**DOI:** 10.1371/journal.pone.0108384

**Published:** 2014-10-07

**Authors:** Charlotte J. Houldcroft, Velislava Petrova, Jimmy Z. Liu, Dan Frampton, Carl A. Anderson, Astrid Gall, Paul Kellam

**Affiliations:** 1 Wellcome Trust Sanger Institute, Wellcome Trust Genome Campus, Hinxton, Cambridge, United Kingdom; 2 Division of Biological Anthropology, Department of Archaeology and Anthropology, University of Cambridge, Cambridge, United Kingdom; 3 Department of Infection, Division of Infection and Immunity, University College London, London, United Kingdom; Helmholtz Zentrum München, Germany

## Abstract

Lymphoblastoid cell lines (LCLs) are commonly used in molecular genetics, supplying DNA for the HapMap and 1000 Genomes Projects, used to test chemotherapeutic agents, and informing the basis of a number of population genetics studies of gene expression. The process of transforming human B cells into LCLs requires the presence of Epstein-Barr virus (EBV), a double-stranded DNA virus which through B-cell immortalisation maintains an episomal virus genome in every cell of an LCL at variable copy numbers. Previous studies have reported that EBV alters host-gene expression and EBV copy number may be under host genetic control. We performed a genome-wide association study of EBV genome copy number in LCLs and found the phenotype to be highly heritable, although no individual SNPs achieved a significant association with EBV copy number. The expression of two host genes (*CXCL16* and *AGL*) was positively correlated and expression of *ADARB2* was negatively correlated with EBV copy number in a genotype-independent manner. This study shows an association between EBV copy number and the gene expression profile of LCLs, and suggests that EBV copy number should be considered as a covariate in future studies of host gene expression in LCLs.

## Introduction

Epstein-Barr virus (EBV) is a ubiquitous human gammaherpesvirus. Following primary infection EBV establishes lifelong persistent infection through latent infection of memory B cells where the virus genome is transcriptionally silent [1], [2]. Reactivation from latency is required for the production of infectious EBV, with such lytic EBV replication being under the control of host and virus factors. In particular, terminal differentiation of memory B cells into plasma cells can lead to EBV lytic reactivation [3]. The mechanisms of host induction of EBV lytic replication are incompletely understood, but periodic shedding of EBV in saliva [4] and variation in saliva virus load between people [5] suggest host genetic variation may contribute to EBV lytic cycle induction. Lymphoblastoid cell lines (LCLs) are human B cells immortalised *in vitro* by EBV and are a useful model of latent infection of B cells. Previous studies on LCLs have shown that when multiple LCLs are derived from the same individual, inter-individual variation in EBV copy number in LCLs is greater than intra-individual variation [6]. A study of the impact of EBV copy number on the gene expression profiles of 198 HapMap LCLs reported that expression of 125 human genes was significantly correlated with EBV copy number [7]. A comparison of Epstein-Barr virus copy number in 62 adult and paediatric LCLs found considerable inter-individual variation in EBV copy number that correlated with expression of immediate-early viral lytic genes BRLF1 and BZLF1, suggesting that spontaneous lytic reactivation is the cause of high EBV genome copy numbers in a subset of LCLs. After the addition of acyclovir, a drug which inhibits viral reactivation, Davies *et al.* showed EBV genome copy numbers fall in LCLs, and return to previous high levels after the removal of acyclovir [8]. This suggests that spontaneous lytic reactivation may be under the control of cell-intrinsic factors. When the viral gene expression profiles of LCLs were compared, using RNAseq data from multiple experiments from different laboratories, Arvey *et al.* [9] reported two major EBV gene expression profiles: latency type III and a lytic pattern of expression. There is evidence of both BZLF1 expression and virus particle production in some LCLs [10]. We have therefore hypothesised that high EBV copy number in LCLs is the result of poor host cell control of the EBV latent-lytic cycle switch, and may be under the control of host genetic factors.

Genome-wide association studies have been successfully used to identify the host genes involved in the pathogenesis of infectious disease [11], [12], [13], [14], [15]. Two genome-wide association studies of genetic control of antibodies to herpesviruses have been performed. A study of EBV antibody titres in ∼2000 individuals identified 15 loci exceeding genome-wide significance associated with either the quantitative or discrete trait of antibody titre [16]. By contrast, a similarly-sized study of cytomegalovirus (CMV) antibody response, a betaherpesvirus which also establishes lifelong latent infection in humans, did not find any genome-wide significant associations [17]. Other studies of host genetic response to herpesvirus infection and lytic reactivation have been limited to family linkage [18] and candidate gene [19] studies of herpes simplex virus-induced disease, and small studies of susceptibility to infection of chickens with an avian herpesvirus, Marek's disease virus [20], [21]. As yet, there have been no attempts to characterise common human genetic polymorphisms associated with cell-intrinsic response to EBV infection.

Here we describe a study to identify human genetic variants associated with Epstein-Barr virus genome copy number in the HapMap [22] and 1000 Genomes [23] LCLs, incorporating sequencing and genotyping data from the HapMap and 1000 Genomes projects. We also investigate differences in gene expression associated with EBV genome copy number using publicly available gene expression data for a subset of the HapMap 3 samples [24].

## Results

### Relative EBV copy number in LCLs and between-population comparisons

**Figure 1:**
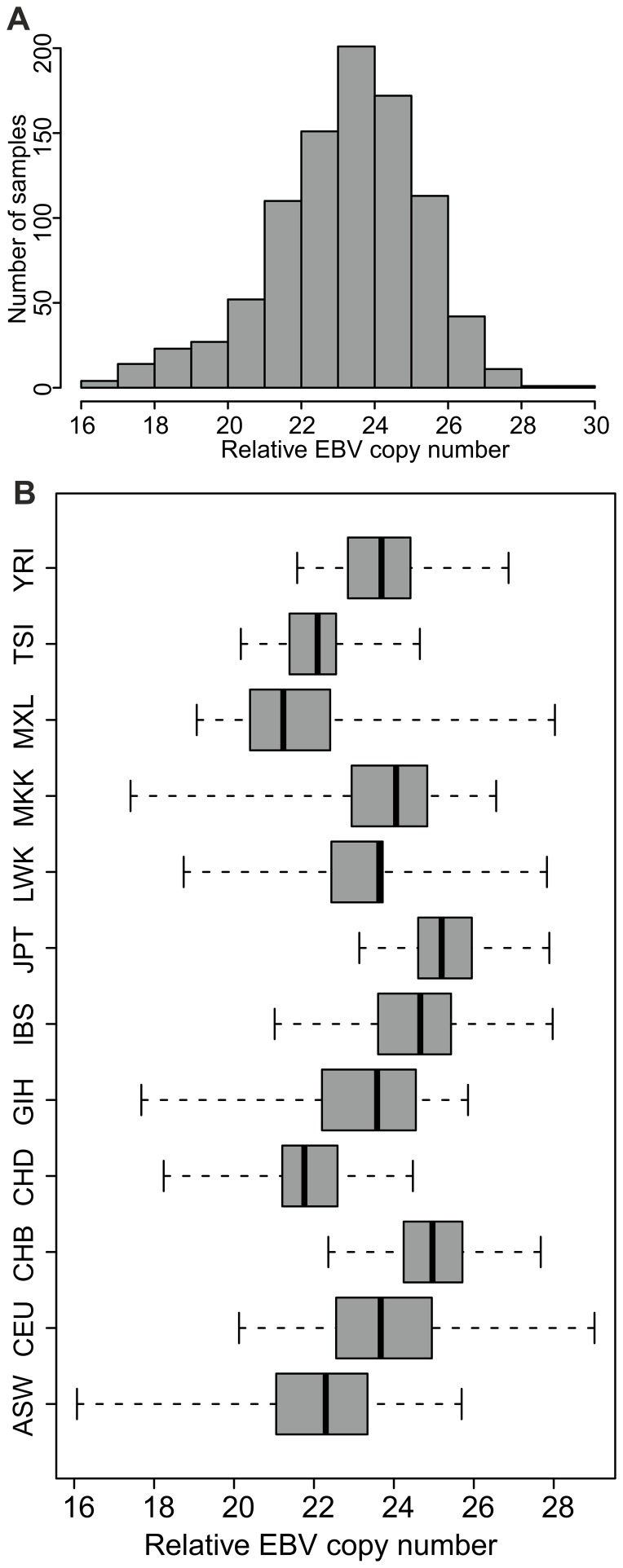
Distribution of relative EBV copy number in LCLs from 12 populations. **A**. Relative EBV copy number in 915 LCLs from 12 populations, including 97 parent-offspring trios. Range 16.07–29.02, mean 23.36 (SD±2.03). The data are not normally distributed (Shapiro-Wilk: W = 0.977, p-value  = 2.086^−11^). **B**. Box and whisker plots showing the range (dotted lines), interquartile range (coloured boxes) and mean relative EBV copy number (central vertical lines) for the 12 populations included in this analysis.

We determined the relative EBV genome copy number for 915 LCLs from the HapMap and 1000 Genomes populations using quantitative PCR (qPCR). The qPCR assay had an 8 log_10_ dynamic range from 1×10^9^ to 1×10^2^ copies/reaction (Figure S1) and an analytical sensitivity of 100 copies/reaction. The PCR efficiency was 99.4%. The samples assayed included individuals from 12 populations (Table 1); and EBV copy numbers of all samples were within the dynamic ranged of the qPCR. Across all populations the mean relative (to single copy host gene) EBV copy number per LCL is 23.36 with a range of 16.07–29.02 (SD±2.03), corresponding to an absolute range of 1 copy per cell to 350 copies per cell (Figure 1 A). We examined the trait of EBV copy number in different populations (Figure 1 B), and found significant differences in the mean EBV copy number between the populations (ANOVA p<2.2×10^−16^). Interestingly, apparently similar ethnic groups in different geographical areas have different mean EBV genome copy numbers. The Denver Han Chinese (CHD) and Beijing Han Chinese (CHB) populations have statistically significantly different means (difference = −3.23, 95% CI −4.34–−2.12), p = 1.17×10^−8^), although the CHB and Japanese from Tokyo (JPT) do not differ from one another significantly. Some European ancestry populations also differ from one another in their mean EBV copy number: Toscani from Italy (TSI) differ from CEU (European ancestry in Utah, USA) (difference  = −1.63, 95% CI −2.52–−0.74, p = 2×10^−7^).

**Table 1:** Samples used in this study.

Population code	Population description*	N (samples)	N (trios)	N (duos)
ASW	People with African Ancestry in the Southwest United States	89	13	21
CEU	Utah residents with ancestry from Northern and Western Europe	84	27	
CHB	Han Chinese in Beijing, China	44		
CHD	Han Chinese in Denver, Colorado	55		
GIH	Gujarati Indians in Houston, Texas	90	1	
IBS	Iberian Populations in Spain	99	1	
JPT	Japanese in Tokyo, Japan	44		
LWK	Luhya in Webuye, Kenya	5		
MKK	Maasai in Kinyawa, Kenya	140	21	5
MXL	People with Mexican Ancestry in Los Angeles, California	92	24	
TSI	Toscani in Italia	67		
YRI	Yoruba in Ibadan, Nigeria	90	30	

*Population names from 1000 Genomes [48] and HapMap [22].

### Association testing

Using the entire sample set we performed a genome wide association study for common host genetic variants associated with LCL EBV copy number. To determine if the EBV that immortalised the LCLs was the laboratory strain B95.8 rather than outgrowth of spontaneous LCLs with wild type EBV we assembled the EBV genomes from 77 CEU and YRI LCLs. We looked for the presence of the deletion specific for the B95.8 genome, which was present in every LCL studied. The frequency of spontaneous LCLs containing wild type EBV, and/or LCLs immortalised with B95.8 and co-infected with wild type EBV, is therefore less than 1 in 77.

**Figure 2:**
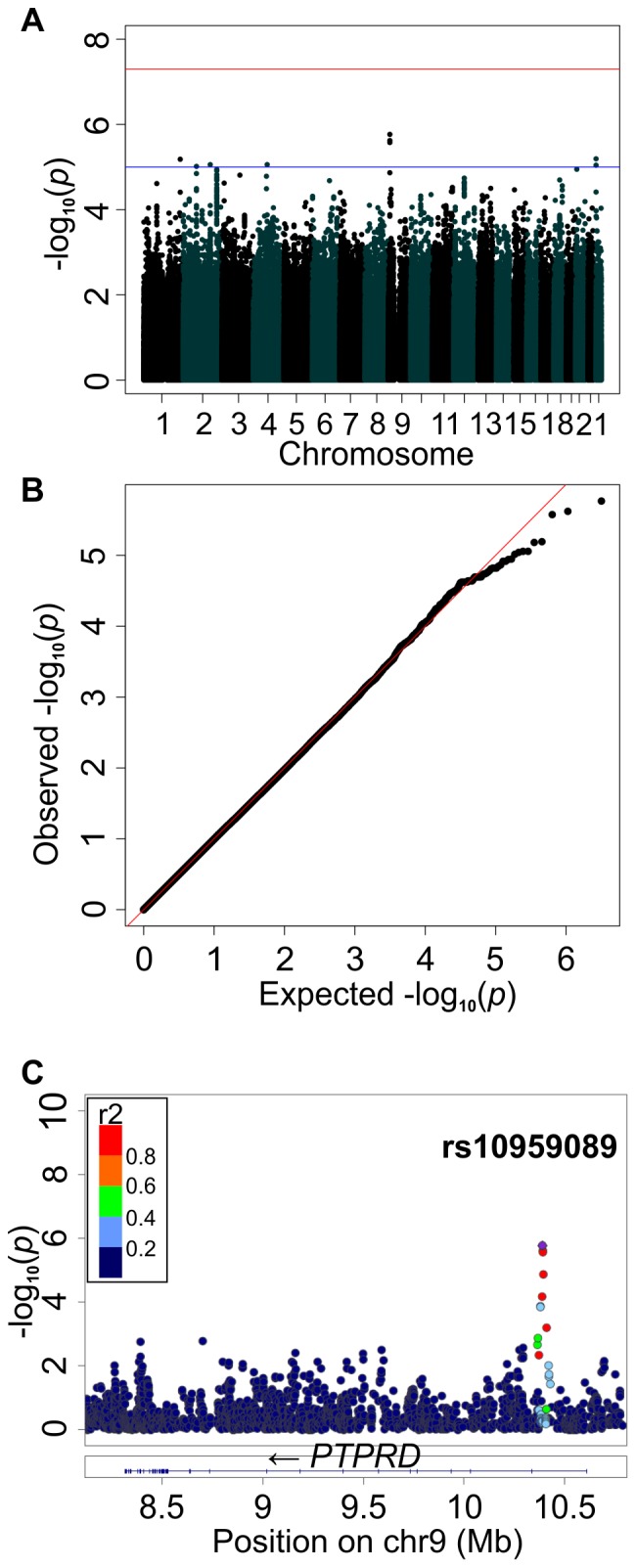
Manhattan plots of the association between EBV copy number and human genetic variants in LCLs. **A**. Results of the stage 2 GWAS of relative EBV copy number in 899 LCLs, derived from 12 populations. In total, ∼1.6 M SNPs, polymorphic in every population studied, were analysed using FaST-LMM. Each point represents a SNP. There were no SNPs with genome-wide significant p-values after correction for multiple testing. **B**. QQ plot showing the distribution of observed test statistics plotted against the expected (null) distribution (red line). The SNPs which fall below the red line suggest that the GWAS may be losing power to detect SNPs associated with EBV copy number. **C**. The strongest signal is located within an intron of protein tyrosine phosphatase receptor delta (*PTPRD*), SNP rs10959089 (p = 1.17×10-6). Plot C made with LocusZoom [56].

After sample-level quality control, genome-wide sequence and genotype data were available for 899 samples from the 1000 Genomes Phase I and HapMap Phase III consensus releases. Mixed-effects modelling [25] was used to test each variant individually for association with EBV copy number in LCLs. Samples without full human genome sequence data were imputed using 1000 Genomes information [26]. Variants were taken forward to association analysis if they were observed to vary in all 12 populations studied with a frequency of >1% and a minimum imputation quality of 0.9. This created a set of 1.6×10^6^ SNPs in common between the samples. The sample size of phenotyped LCLs with genotype or whole genome sequence information available is 899 individuals, including 798 unrelated individuals, once offspring of 101 trios were removed. The statistical power of the study to detect a variant explaining a given proportion of the total trait variance was calculated using GWApower [27]. Our study had an 80% power to detect a variant explaining 4.7% of the total variance in relative EBV copy number. The overall distribution of P values showed little evidence of genomic inflation (λ = 0.98), consistent with the null hypothesis and suggesting that mixed-effects modelling was able to correct for high familial relatedness and population structure (Figure 2 A and B).

We estimated the proportion of variance in EBV copy number within the dataset explained by the set of ∼1.6 million common genetic variants using GCTA, in 677 unrelated individuals to be 0.65 (SE ±0.38; p = 0.04). We also calculated the heritability of EBV copy number in 101 trios present within the 1000 Genomes dataset, where EBV copy number was available for all trio members. The parental mid-point EBV copy number was regressed against the offspring EBV copy number, giving a heritability estimate of 34% (SE ±11, p = 0.0028).

No variants passed the genome-wide significance threshold (P<5×10^−8^, Figure 2 A), although 98 SNPs achieved a genome wide significance of <1×10^−5^ and are suggestive of a possible association (Table S2 in File S1). Of these the top SNP, rs10959089 (P = 1.17×10^−6^, beta  = 0.52 for the minor C allele), was located in the first intron of the gene *PTPRD* (protein tyrosine phosphatase receptor delta) (Figure 2 C).

### Association testing of variants implicated in EBV infection, immune response and disease by previous studies

48 SNPs and small structural variants have been previously reported to influence EBV traits such as acquisition risk, antibody response, or EBV-positive disease risk. 28 of these SNPs were included in our association study (Table 2). Two SNPs had P values of nominal significance (rs2516049, p = 0.01; rs1052536, p = 0.03). It is therefore not possible to link these variants to the phenotype of relative EBV copy number in LCLs.

**Table 2:** Results for 27 SNPs previously reported to be associated with EBV infection, immune response or EBV-positive disease.

SNP	Gene	Minor allele	Chr	Position	P value	Beta	Reference
rs17102086	RAD54L	C	1	46722939	0.69	1.05	[49]
rs1801274	FCGR2A	G	1	161479745	0.59	1.05	[50]
rs1800587	IL1A	A	2	113542960	0.47	1.08	[51]
rs2306597	RFC1	A	4	39297200	0.56	1.07	[49]
rs10947261	BTNL2	T	6	32373232	0.55	1.08	[38]
rs10947262	BTNL2	T	6	32373312	0.60	1.07	[38]
rs1800629	TNF	A	6	31543031	0.47	1.14	[52]
rs204999		G	6	32109979	0.75	1.04	[38]
rs2213585	HLA-DRA	G	6	32413150	0.37	1.09	[38]
rs2213586	HLA-DRA	A	6	32413094	0.37	1.09	[38]
rs2227139		G	6	32413459	0.37	1.09	[38]
rs2239803	HLA-DRA	C	6	32411833	0.44	1.08	[38]
rs2294882	BTNL2	C	6	32367515	0.41	1.11	[38]
rs2294884	BTNL2	G	6	32367259	0.54	1.08	[38]
rs2516049	LOC100507709	C	6	32570400	0.01	1.32	[38]
rs28362683	BTNL2	A	6	32372963	0.84	1.03	[38]
rs3130048	BAG6	C	6	31613739	0.62	1.06	[38]
rs4248166	BTNL2	C	6	32366421	0.75	1.04	[38]
rs477515	LOC100507709	A	6	32569691	0.04	1.25	[38]
rs652888	EHMT2	G	6	31851234	0.15	1.19	[38]
rs6904029	HCG9	A	6	29943067	0.86	1.02	[53]
rs7192	HLA-DRA	T	6	32411646	0.37	1.09	[38]
rs7195	HLA-DRA	A	6	32412539	0.37	1.09	[38]
rs9264942	HLA-C	C	6	31274380	0.17	1.14	[54]
rs9268832		T	6	32427789	0.37	1.09	[38]
rs1800450	MBL2	T	10	54531235	0.85	1.03	[55]
rs1052536	LIG3	T	17	33331575	0.03	1.28	[49]

### Epstein-Barr virus gene copy number, host gene expression and eQTL analysis in LCLs

**Figure 3:**
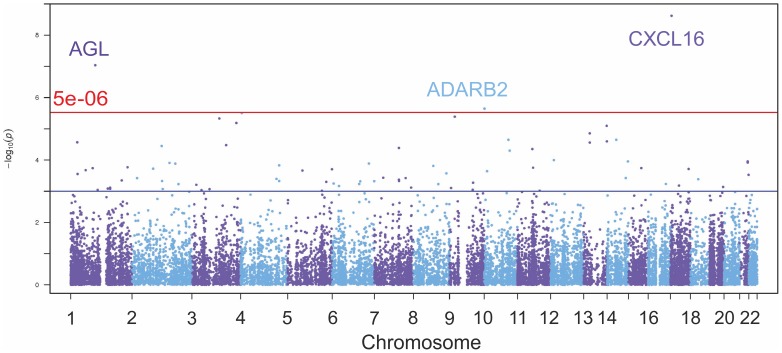
Association between EBV copy number and human gene expression in LCLs. Manhattan plot showing the correlation between expression levels of 21,800 individual gene transcripts from microarray data and EBV copy number.

Microarray gene expression data was available for 466 unrelated individuals from 8 populations [24]. A linear regression was performed for these individuals between 21,800 gene transcripts and EBV genome copy number. A statistically significant positive correlation was found between EBV relative copy number and the expression levels of two genes: *CXCL16* (chemokine (C-X-C motif) ligand 16) and *AGL* (amylo-alpha-1, 6-glucosidase, 4-alpha-glucanotransferase), and a statistically significant negative correlation between EBV relative copy number and *ADARB2* (adenosine deaminase, RNA-specific, B2) expression (Figure 3; Table 3). Transcripts with suggestive P values (P>5×10^−3^) are included in Table S3 in File S1. Evidence for the effect of EBV genome copy number on eQTL results was not observed for any of these genes; the correlation did not occur in a genotype-dependent manner. QTL mapping using EBV as a phenotype did not reveal any statistically significant SNPs located in or near these genes.

**Table 3:** Gene transcripts associated with relative EBV copy number with P<3×10^−5^.

Gene	Array_tag	Chr	Beta	SE	P value
CXCL16	ILMN_1672278	17	0.114	0.019	2.4×10^−9^
AGL	ILMN_1680343	1	0.039	0.007	9.3×10^−8^
ADARB2	ILMN_1749493	10	−0.042	0.009	2.3×10^−6^

## Discussion

This is the largest study to investigate the impact of host genetic factors on Epstein-Barr virus genome copy number in lymphoblastoid cell lines. As inhibitors of the EBV lytic cycle effectively reduce EBV copy number in LCLs this suggests the higher copy numbers are the result of lytic replication induction [8]. Our study therefore also represents a proxy phenotype for the spontaneous switch from latent to lytic EBV replication in LCLs. We identified the EBV strain infecting a sample of the LCLs, quantified relative EBV genome copy number in 915 LCLs from the HapMap and 1000 Genomes study, performed a genome-wide association study of EBV genome copy number, estimated the heritability of EBV genome copy number in parent-offspring trios, and examined the relationship between EBV genome copy number and human gene expression in a subset of LCLs.

Using high-coverage sequence data from the 1000 Genomes Pilot project for 77 CEU and YRI LCLs, we estimate that the rate of LCLs containing wild-type or non-B-95.8-strain EBV is less than 1 in 77. This is consistent with the findings of Santpere and colleagues [25], who detected unambiguous evidence of EBV co-infection or wild-type infection in 10 out of 929 LCLs from the 1000 Genomes Project dataset. These 10 LCLs were not included in our analysis, therefore competition between viral strains was unlikely to be a factor influencing copy number in our study.

Our results, of relative EBV copy number varying between individuals, and within and between populations are consistent with previous studies [6], [8] of LCLs of unreported ancestry, where EBV genome copy number was a trait that varied more significantly between LCLs than between different passages or sub-cultures of the same LCL. Davies *et al.* [8] suggest that the variation in EBV copy number is controlled by cell-intrinsic factors. In our analysis, it remained unclear whether genetic or cell-intrinsic factors caused the differences in mean EBV copy number between the Denver Han Chinese (CHD) and Beijing Han Chinese (CHB) populations. While it has been noted that the CHD LCLs grow more slowly than the Asian (ASN) LCLs (which include the CHB LCLs) [28], another study [29] found no statistically significant relationship between LCL growth rate and EBV copy number. We conclude that although cell intrinsic factors are still the most plausible explanation, multiple genetic variants, each with a small effect of EBV copy number, also likely play a role.

The strongest association signal in the genome-wide association study was an intronic SNP, located in gene *PTPRD*. *PTPRD* functions in cellular signalling, is located on the cell surface, and has been identified as a tumour suppressor. Mutations within *PTPRD* have been associated with several cancers: glioblastoma multiforme and head and neck squamous cell carcinomas [30], and clear cell renal carcinoma [31]. The paralogous gene *PTPRK* (protein tyrosine phosphatase receptor kappa) interacts with EBV. When *PTPRK* is over-expressed in EBV-infected Hodgkin lymphoma cells, survival of these cells decreases; when *PTPRK* expression is knocked down by RNAi, survival increases; suggesting a role for *PTPRK* in tumour suppression. The EBV gene EBNA1 targets Smad2, a protein that regulates *PTPRK* expression. By decreasing the half-life of Smad2, *PTPRK* is down-regulated in turn [32]. *PTPRC* (protein tyrosine phosphatase receptor C) has recently been associated with herpes simplex encephalitis susceptibility in mice [33]. Therefore although not genome wide significant, polymorphisms in *PTPRD* are in a biologically plausible gene.

Changes in the relative EBV copy number of LCLs have an impact on the gene expression profiles of those LCLs in a genotype-independent manner. Our analysis of genes differentially expressed between LCLs identified three genes whose expression positively (*CXCL16* and *AGL*) or negatively (*ADARB2*) correlated with relative EBV copy number. *CXCL16* is regulated by the microRNA CMVmiR-M23-2 of another human herpesvirus, CMV [34]. Reptar [35] predicts that *CXCL16* is targeted by seven EBV microRNAs (ebv-miR-BART17-5p, ebv-miR-BART21-3p, ebv-miR-BART21-5p, ebv-miR-BART22, ebv-miR-BART3*, ebv-miR-BART5* and ebv-miR-BART7). It is also interesting to note that *CXCL16* is a chemokine which has recently been associated with disease activity in multiple sclerosis [36] and mouse models of experimental autoimmune encephalomyelitis [37]. EBV microRNAs are also predicted to interact with *AGL* and *ADARB2* [35]. It is likely that in a larger sample of LCLs, further genes would show significant correlations with relative EBV copy number. It is therefore important to control for the effects of EBV copy number in gene expression studies utilising large samples of LCLs. Other studies of the impact of EBV on the gene expression patterns of LCLs include a study by Choy *et al.*, which reported that ∼15% of genes to have at least 5% of their variance correlated with EBV copy number [7].

In a study of semi-quantitative antibody response to EBV gene EBNA1, Rubicz *et al.* [38] were able to identify 15 SNPs which were associated with EBV antibody response at genome-wide significant level. In contrast, Kuparinen *et al.* [17] performed a GWAS for CMV antibody response and did not identify any genome-wide significant SNPs. It is possible that a different genetic architecture underlies the host response to control of EBV copy number in LCLs than that of EBV antibody response. Additionally all of the genes associated with the EBV antibody response were within the HLA system, a region of the genome this study was not well-powered to interrogate given the differences in HLA allele frequencies between populations and our criteria that a variant must be present in all 12 populations studied for SNP inclusion. Our finding that the 1.6 M SNPs studied in this GWAS could collectively explain 65% (se = 38%) of the variance in relative EBV copy number, while no single SNP reached genome-wide significance, suggests that many variants of small effect play a collective role within LCLs. Similarly, GWAS of genetic resistance to HIV-1 infection [15] could not find any common variants (with the exception of CCR5-delta 32) which were protective, while GWAS of host control of HIV-1 viral load found a number of genome-wide significant loci associated with lower HIV-1 viral load set points and slower progression to AIDS [12], [39]. The power to identify host genetic variation of virus infection traits may greatly depend on the trait under study and the sample sizes.

We estimated the heritability of relative EBV copy number, based on data from 101 parent-child trios to be 34% (se ±11%, p = 0.003). Other studies have found EBV anti-EBNA1 antibody response to be 68% heritable when considered as a discrete trait (seropositive versus seronegative) [38] and discrete anti-VCA IgG antibody response to be 32–48% heritable [40]. Infectious mononucleosis concordance rates in twins were estimated to be 12% between monozygotic twins and 6% between dizygotic twins [41]. Therefore, host genetic factors appear to play a variable but significant role in symptomatic response to primary EBV infection, the adaptive immune response to EBV latency, and the cell-intrinsic control of EBV latency although none of the variants previously identified were significantly associated with EBV copy number in our analysis.

This study has established that relative EBV copy number within LCLs is very much a complex trait, which has a significant heritable component. Our results suggest that many genetic variants of effect size less than 4.7% of variance in relative EBV copy number exist, but which this study was not statistically powered to detect. This study also focused on 1.6 million single nucleotide polymorphisms which were common to every population studied. Because SNPs which did not vary in all 12 populations studied were excluded from this analysis due to potential population stratification, we cannot rule out the effect on relative EBV copy number of SNPs which were only variable in a subset of the populations studied. We also cannot exclude the effects of rare variants of large effect on relative EBV copy number in LCLs, as these were not studied here. It is also possible that structural variants which are poorly tagged by common SNPs may play a role. To identify the genetic factors that underpin EBV copy number, a significant increase in sample size is necessary that will become possible within on-going large-scale sequencing and genotyping projects. However, we do find that even in a relatively modest sample size, EBV copy number is correlated with LCL host-gene expression patterns, in a host genotype-independent manner. Studies using larger samples of LCLs to study host gene expression profiles may find EBV-associated changes in LCLs generate false-positive results, unless EBV copy number is controlled for.

## Methods

### Ethics

No primary human tissue was used in this study. Details of this project were sent to the Coriell Cell Repository to be passed on the relevant Community Advisory Groups for HapMap participants.

### Samples

Separated from peripheral blood as part of the HapMap and 1000 Genomes Projects, LCLs and LCL-derived DNA were provided to Wellcome Trust Sanger Institute by the Coriell Cell Repository. DNA from 915 HapMap lymphoblastoid cell lines was obtained from Coriell Institute for Medical Research, representing 12 populations. A summary of the composition of the sample group that provided the LCLs is provided in Table 1. Cell line BCBL-1 was used as a calibrator for quantitative PCR. It is a Kaposi Sarcoma Herpesvirus-positive, EBV-negative body cavity-derived primary effusion lymphoma cell line, of B cell origin [42].

### Quantification of relative EBV copy number per cell

Quantification of relative EBV copy number was performed using quantitative PCR (qPCR). An artificial gene (GeneArt, Life Technologies) was designed based on the EBV BALF5 sequence for use as a positive control in qPCR, but containing an artificially inserted sequence to distinguish it from wild-type BALF5, with the sequence: *CCCTGTTTATCCGATGGAATGACGGCGCATTTCTCGTGCGTGTACACCGTCTCGAGTATGACTGGTTCCAATTGACAAGCTGGGTCGTAGACATGGAAGTCCAGAGGGCTTCCG*. Quantitative PCR was performed on an Agilent MxPro 3005 machine using the QuantiTect Multiplex PCR NoROX Kit (Qiagen). The PCR reagents were: 2.5 µl nuclease-free water (Qiagen), 12.5 µl QuantiTect Multiplex PCR No ROX mastermix (Qiagen), 2 µl BALF5 primer mix (10 pmol/µl of each primer and 0.75 pmol/µl probe [43]), 2 µl GAPDH primer mix (2.5 pmol/µl of each primer and 2 pmol/µl probe [44]), 1 µl ROX (diluted 1∶10 in 10 mM Tris-HCL (Life Technologies)), and 5 µl template DNA or positive control. EBV qPCR primer and probe sequences were from Kimura, 1999 [43]. GAPDH primer and probe sequences were from Pardieu, 2010 [44]. PCR conditions were as follows: 95°C for 15 minutes, followed by 45 cycles of 94°C for 60 seconds, 57°C for 30 seconds and 72°C for 30 seconds. Fluorescence data was collected during the annealing step.

The 2−ΔΔCT method [45] was used for relative quantification of target gene abundance (target gene BALF5, endogenous control gene *GAPDH*). Gene copy numbers in LCLs were normalised against the BCBL-1 cell line [46]. Data analysis was performed using MXPro v4.10 qPCR software (Agilent Technologies).

### Heritability analysis

Within the individuals with relative EBV copy number data available, there were 101 trios with EBV copy number information available for mother, father and offspring. The family structure was taken from the 1000 Genomes pedigree files. They were drawn from four populations - CEU, IBS, MXL and YRI. The mid-parental average for the phenotype (relative EBV copy number) was calculated and the child's phenotype regressed against the mid-parental phenotype. The regression gives an estimate of narrow sense heritability of the trait and its associated P value.

### Genotyping

899 samples have been sequenced or genotyped using three different platforms. For 355 samples, sequencing data was available from the 1000 Genomes Project Phase 1 release (http://ftp.1000genomes.ebi.ac.uk/vol1/ftp/phase1/analysis_results/integrated_call_sets/); for 313 samples, genotypes was available from the 1000 Genomes Project Illumina HumanOmni2.5–Quad v1-0 B_SNP data release (ftp://ftp.1000genomes.ebi.ac.uk/vol1/ftp/technical/working/20120131_omni_genotypes_and_intensities/); and for 231 samples, genotypes were available from the HapMap Phase III consensus release (ftp://ftp.ncbi.nlm.nih.gov/hapmap/genotypes/2009-01_phaseIII/plink_format/). Where multiple sources of genotyping or sequencing data were available for a sample, 1000 Genomes Phase I sequence data was used in preference to other sources, followed by Illumina 2.5 M Omni genotypes, and finally HapMap Phase III genotypes. Briefly, quality control for SNP and sample inclusion was as follows: Hardy-Weinberg equilibrium *P* value of >1×10^−6^; minor allele frequency of >1%; SNP call rate of >95%; and a sample call rate of >95%. Monomorphic SNPs were excluded. All quality control was performed using PLINK [47].

### Imputation and association testing

The statistical power to identify a genetic variant was calculated using GWApower [27]. Imputation of genotyped samples to 1000 Genomes Phase 1 was performed using IMPUTE2 [26]. This increased the number of SNPs in common between the samples from 600,000 to 37 million. These imputed variants were then subjected to quality control in PLINK [47], namely removing: SNPs where Hardy-Weinburg equilibrium was P<1×10^−5^ in at least one of the 1000 Genomes populations (n = 8,754,733); SNPs which were monomorphic among phenotyped samples (n = 8,689,910); SNP with a missingness >0.01 (n = 9,808,778) and all SNPs with MAF <0.01 (n = 16,881,983). SNPs which were called discordantly in samples where sequencing and genotyping information were both available were excluded from further analysis. As EBV copy number varies significantly between different populations, we performed additional QC in order to account for potential stratification in the association analysis: removing any SNP if HWE P<0.01 in two or more populations, and removing any SNP that was monomorphic in at least one of the populations. This left a final set of 1,595,489 SNPs which were polymorphic in every population studied. Association analysis was performed using a linear mixed model implemented in FaST-LMM-Select [25].

### Transcriptional profiles

Microarray expression data from Stranger *et al.* [24] (“REDUCED” dataset) was obtained for 466 unrelated individuals with relative EBV copy numbers. Expression data is available on http://www.ebi.ac.uk/arrayexpress/(Series Accession Number E-MTAB-264 and E-MTAB-198.processed.1). For each individual, correlation between expression levels of individual gene transcripts and EBV copy number was determined via linear regression. P-values of correlation with EBV copy number were obtained for each gene transcript (21,800 in total). Transcripts with p-value lower than 5×10^−6^ were considered significantly correlated with EBV copy number. Transcripts were mapped back to the genes they correspond to according to the array design file (“A-MEXP-930.adf.txt”) available on http://www.ebi.ac.uk/arrayexpress/experiments/E-MTAB-264/

### eQTL analysis

Effect of EBV relative copy number on the identification of expression quantitative loci (eQTLs) was tested in PLINK via linear association model with or without EBV copy number used as a covariate. Expression data included normalized log_2_ quantitative gene expression measurements for the 21,800 probes (18,226 unique autosomal genes) from 466 unrelated individuals of HapMap Phase III assayed on the Illumina Sentrix Human-6 Expression BeadChip [24]. SNP genotypes were as described for the QTL analysis. SNPs within a 2 Mb window around a gene locus were defined as eQTLs. Correction for population structure was performed using principal component analysis (PCA). Difference in effect sizes of eQTLs identified in the two association studies (with or without EBV copy number as a covariate) was determined via paired t-test across all tested SNPs (1.6 million). Resulting p-values of the difference in effect sizes were plotted for each transcript across all 22 non-sex chromosomes.

## Supporting Information

Figure S1 **Standard curve of BALF5 quantitative PCR primer set dilution series, which amplifies the EBV polymerase gene BALF5.** The efficiency of the BALF5 qPCR assay was 99.4% (compared to GAPDH), r2 = 0.998.
(DOCX)

File S1 **Supplementary tables.** Table S1 in File S1 shows the results of a Tukey honest significant difference test of mean EBV copy number between populations. Table S2 in File S1 summarises the results of the EBV copy number GWAS, including all SNPs associated with EBV copy number with P<5×10^−5^. Table S3 in File S1 shows changes in gene expression (microarray) correlated with EBV copy number with suggestive P values (P >5×10^−3^).
(DOCX)

File S2 **Spreadsheet of EBV copy number data for HapMap and 1000 Genomes LCLs included in this study.**
(XLSX)
