# Supplementary material for: Host Genetic Variants and Gene Expression Patterns Associated with Epstein-Barr Virus Copy Number in Lymphoblastoid Cell Lines
Source: PLoS One. 2014 Oct 7;9(10):e108384. doi: 10.1371/journal.pone.0108384 (PMC4188571; doi:10.1371/journal.pone.0108384)
Supplement: Figure S1 — Standard curve of BALF5 quantitative PCR primer set dilution series, which amplifies the EBV polymerase gene BALF5. The efficiency of the BALF5 qPCR assay was 99.4% (compared to GAPDH), r2 = 0.998. (DOCX) [file pone.0108384.s001.docx]

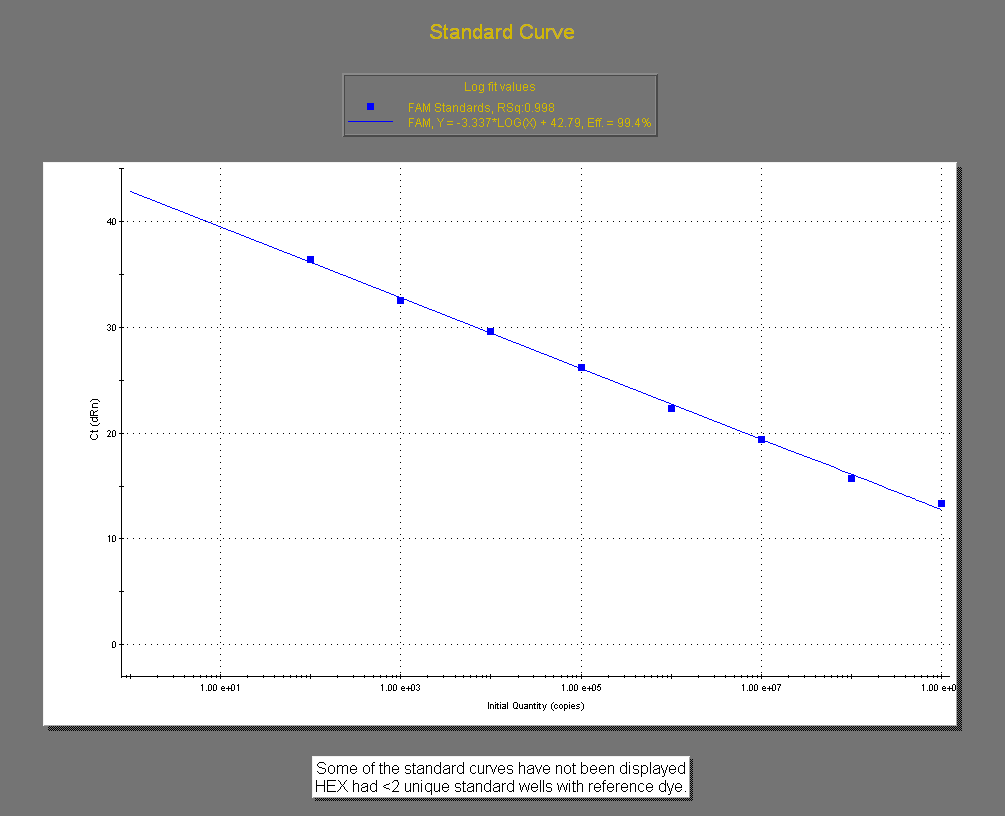


Figure S1: Standard curve for BALF5 qPCR

Standard curve of BALF5 primer set dilution series, which amplifies the EBV polymerase gene BALF5. The efficiency of the BALF5 qPCR assay was 99.4% (compared to *GAPDH*), with an EBV BALF5 standard series. r^2^ = 0.998
