# Supplementary material for: Host Genetic Variants and Gene Expression Patterns Associated with Epstein-Barr Virus Copy Number in Lymphoblastoid Cell Lines
Source: PLoS One. 2014 Oct 7;9(10):e108384. doi: 10.1371/journal.pone.0108384 (PMC4188571; doi:10.1371/journal.pone.0108384)
Supplement: File S1 — Supplementary tables. Table S1 in File S1 shows the results of a Tukey honest significant difference test of mean EBV copy number between populations. Table S2 in File S1 summarises the results of the EBV copy number GWAS, including all SNPs associated with EBV copy number with P<5×10^−5^. Table S3 in File S1 shows changes in gene expression (microarray) correlated with EBV copy number with suggestive P values (P >5×10^−3^). (DOCX) [file pone.0108384.s002.docx]

**SUPPLEMENTARY TABLES**

We performed a Tukey ‘Honest Significant Difference’ test of the means in R, a multi-way test of which population means differ from one another, corrected for multiple testing. The significance threshold was set at α = 0.05 (table 4.2).

**Table S1: Tukey honest significant difference test of mean EBV genome load between populations**

|  | **Difference** | **Lower CI** | **Upper CI** | **Adjusted p-value** |
| --- | --- | --- | --- | --- |
| **CEU-ASW** | 2.137022 | 1.295727 | 2.978318 | 0† |
| **CHB-ASW** | 3.413066 | 2.386517 | 4.439615 | 0 |
| **CHD-ASW** | 0.184288 | -0.75317 | 1.121745 | 0.9999666 |
| **GIH-ASW** | 1.3609 | 0.521944 | 2.199855 | 0.0000087 |
| **IBS-ASW** | 2.906022 | 2.086247 | 3.725798 | 0 |
| **JPT-ASW** | 3.608228 | 2.57396 | 4.642496 | 0 |
| **LWK-ASW** | 1.637955 | -0.94141 | 4.217319 | 0.6368118 |
| **MKK-ASW** | 1.844765 | 1.091008 | 2.598521 | 0 |
| **MXL-ASW** | 0.072096 | -0.76231 | 0.906505 | 1 |
| **TSI-ASW** | 0.504103 | -0.38547 | 1.393674 | 0.7855602 |
| **YRI-ASW** | 2.099288 | 1.260333 | 2.938244 | 0 |
| **CHB-CEU** | 1.276044 | 0.249494 | 2.302593 | 0.0029329 |
| **CHD-CEU** | -1.95273 | -2.89019 | -1.01528 | 0 |
| **GIH-CEU** | -0.77612 | -1.61508 | 0.062832 | 0.1013091 |
| **IBS-CEU** | 0.769 | -0.05078 | 1.588775 | 0.0902182 |
| **JPT-CEU** | 1.471205 | 0.436937 | 2.505473 | 0.0002266 |
| **LWK-CEU** | -0.49907 | -3.07843 | 2.080296 | 0.9999716 |
| **MKK-CEU** | -0.29226 | -1.04601 | 0.461498 | 0.9825399 |
| **MXL-CEU** | -2.06493 | -2.89934 | -1.23052 | 0 |
| **TSI-CEU** | -1.63292 | -2.52249 | -0.74335 | 0.0000002 |
| **YRI-CEU** | -0.03773 | -0.87669 | 0.801221 | 1 |
| **CHD-CHB** | -3.22878 | -4.33551 | -2.12205 | 0 |
| **GIH-CHB** | -2.05217 | -3.0768 | -1.02753 | 0 |
| **IBS-CHB** | -0.50704 | -1.51603 | 0.501944 | 0.8912126 |
| **JPT-CHB** | 0.195162 | -0.99469 | 1.385008 | 0.9999948 |
| **LWK-CHB** | -1.77511 | -4.4207 | 0.870478 | 0.5511384 |
| **MKK-CHB** | -1.5683 | -2.52443 | -0.61218 | 0.0000063 |
| **MXL-CHB** | -3.34097 | -4.36188 | -2.32006 | 0 |
| **TSI-CHB** | -2.90896 | -3.97543 | -1.84249 | 0 |
| **YRI-CHB** | -1.31378 | -2.33841 | -0.28915 | 0.0017312 |
| **GIH-CHD** | 1.176611 | 0.241254 | 2.111968 | 0.0024054 |
| **IBS-CHD** | 2.721734 | 1.803541 | 3.639927 | 0 |
| **JPT-CHD** | 3.423939 | 2.310047 | 4.537832 | 0 |
| **LWK-CHD** | 1.453667 | -1.15864 | 4.065977 | 0.8049781 |
| **MKK-CHD** | 1.660476 | 0.800712 | 2.520241 | 0 |
| **MXL-CHD** | -0.11219 | -1.04347 | 0.819089 | 0.9999998 |
| **TSI-CHD** | 0.319815 | -0.6612 | 1.300825 | 0.9958693 |
| **YRI-CHD** | 1.915 | 0.979643 | 2.850357 | 0 |
| **IBS-GIH** | 1.545123 | 0.727749 | 2.362497 | 0.0000001 |
| **JPT-GIH** | 2.247328 | 1.214963 | 3.279694 | 0 |
| **LWK-GIH** | 0.277056 | -2.30155 | 2.855657 | 0.9999999 |
| **MKK-GIH** | 0.483865 | -0.26728 | 1.235009 | 0.6152188 |
| **MXL-GIH** | -1.2888 | -2.12085 | -0.45675 | 0.0000301 |
| **TSI-GIH** | -0.8568 | -1.74415 | 0.030561 | 0.0699258 |
| **YRI-GIH** | 0.738389 | -0.09822 | 1.574998 | 0.1454268 |
| **JPT-IBS** | 0.702205 | -0.31463 | 1.719046 | 0.5042232 |
| **LWK-IBS** | -1.26807 | -3.84049 | 1.304358 | 0.9034757 |
| **MKK-IBS** | -1.06126 | -1.79092 | -0.3316 | 0.0001382 |
| **MXL-IBS** | -2.83393 | -3.64663 | -2.02122 | 0 |
| **TSI-IBS** | -2.40192 | -3.27117 | -1.53267 | 0 |
| **YRI-IBS** | -0.80673 | -1.62411 | 0.01064 | 0.0568768 |
| **LWK-JPT** | -1.97027 | -4.61887 | 0.678321 | 0.3817388 |
| **MKK-JPT** | -1.76346 | -2.72787 | -0.79906 | 0.0000002 |
| **MXL-JPT** | -3.53613 | -4.56481 | -2.50746 | 0 |
| **TSI-JPT** | -3.10412 | -4.17803 | -2.03022 | 0 |
| **YRI-JPT** | -1.50894 | -2.5413 | -0.47657 | 0.0001235 |
| **MKK-LWK** | 0.20681 | -2.34534 | 2.758963 | 1 |
| **MXL-LWK** | -1.56586 | -4.14298 | 1.011267 | 0.6997336 |
| **TSI-LWK** | -1.13385 | -3.72936 | 1.461658 | 0.957185 |
| **YRI-LWK** | 0.461333 | -2.11727 | 3.039935 | 0.9999872 |
| **MXL-MKK** | -1.77267 | -2.51873 | -1.02661 | 0 |
| **TSI-MKK** | -1.34066 | -2.14794 | -0.53338 | 0.0000044 |
| **YRI-MKK** | 0.254524 | -0.49662 | 1.005667 | 0.9942341 |
| **TSI-MXL** | 0.432007 | -0.45105 | 1.315067 | 0.9079983 |
| **YRI-MXL** | 2.027192 | 1.195143 | 2.859242 | 0 |
| **YRI-TSI** | 1.595185 | 0.707828 | 2.482543 | 0.0000004 |

†Boxes marked in grey are statistically significant different

**Table S2: SNPs associated with EBV genome load with P < 5x10^-5^**

| **SNP** | **Chr** | **BP** | **P value** | **Gene** | **Odds Ratio** | **A1** | **A2** | **Beta**† | **Standard error** |
| --- | --- | --- | --- | --- | --- | --- | --- | --- | --- |
| rs10959089 | 9 | 10391363 | 1.71E-06 | PTPRD | 1.68 | C | G | 0.520912 | 0.108141 |
| rs36063109 | 9 | 10393577 | 2.39E-06 | PTPRD | 1.67 | C | G | 0.515086 | 0.108479 |
| rs745002 | 9 | 10394296 | 2.66E-06 | PTPRD | 1.67 | T | C | 0.511816 | 0.108294 |
| rs73145665 | 22 | 17426414 | 6.41E-06 | GAB4 | 2.04 | C | T | 0.715025 | 0.157513 |
| rs6661407 | 1 | 230654589 | 6.57E-06 | PGBD5 | 1.56 | A | C | -0.44439 | 0.098007 |
| rs55802491 | 4 | 87180196 | 8.76E-06 | MAPK10 | 1.74 | A | T | -0.55263 | 0.123586 |
| rs11893040 | 2 | 170092395 | 8.77E-06 | LRP2 | 1.55 | G | A | -0.4355 | 0.097396 |
| rs5748749 | 22 | 17417742 | 9.06E-06 | GAB4 | 2.02 | T | C | 0.701075 | 0.157047 |
| rs7423195 | 2 | 83513890 | 9.65E-06 | Nothing | 1.57 | A | G | 0.448661 | 0.100814 |
| rs2423247 | 20 | 7378634 | 1.13E-05 | HAO1 | 1.62 | C | T | -0.48453 | 0.109722 |
| rs1458285 | 2 | 211242324 | 1.14E-05 | MYL1 | 1.56 | A | C | -0.4455 | 0.100949 |
| rs1458284 | 2 | 211242258 | 1.21E-05 | MYL1 | 1.56 | A | T | -0.44412 | 0.100929 |
| rs1458286 | 2 | 211242377 | 1.21E-05 | MYL1 | 1.56 | G | C | -0.44412 | 0.100929 |
| rs10959090 | 9 | 10395912 | 1.36E-05 | PTPRD | 1.57 | A | G | 0.453339 | 0.103636 |
| rs11126889 | 2 | 83386258 | 1.42E-05 | Nothing | 1.65 | T | G | 0.501222 | 0.114833 |
| rs1458287 | 2 | 211242700 | 1.51E-05 | MYL1 | 1.55 | G | A | -0.43948 | 0.101007 |
| rs1350465 | 2 | 211242811 | 1.51E-05 | MYL1 | 1.55 | T | C | -0.43948 | 0.101007 |
| rs1350466 | 2 | 211242989 | 1.51E-05 | MYL1 | 1.55 | T | A | -0.43948 | 0.101007 |
| rs13070953 | 3 | 113318748 | 1.55E-05 | SIDT1 | 1.67 | G | A | 0.515302 | 0.118586 |
| rs4392506 | 4 | 82645405 | 1.65E-05 | RASGEF1B | 1.62 | T | A | 0.480858 | 0.111007 |
| rs16844361 | 2 | 211242053 | 1.72E-05 | MYL1 | 1.55 | G | T | -0.43635 | 0.100953 |
| rs62202077 | 2 | 211239260 | 1.78E-05 | MYL1 | 1.55 | A | T | -0.43732 | 0.101364 |
| rs12581953 | 12 | 66908808 | 1.82E-05 | GRIP1 | 1.52 | A | G | 0.416093 | 0.096564 |
| rs34583648 | 12 | 66900305 | 1.83E-05 | GRIP1 | 1.53 | T | C | 0.422265 | 0.098021 |
| rs62202079 | 2 | 211239622 | 1.89E-05 | MYL1 | 1.55 | T | C | -0.43544 | 0.101238 |
| rs2043186 | 18 | 36946528 | 2.01E-05 | LINC00669 | 1.65 | A | G | -0.49864 | 0.116315 |
| rs57726246 | 2 | 211238605 | 2.02E-05 | MYL1 | 1.54 | C | T | -0.43372 | 0.1012 |
| rs61479752 | 2 | 211238883 | 2.02E-05 | MYL1 | 1.54 | G | A | -0.43372 | 0.1012 |
| rs62202075 | 2 | 211239001 | 2.02E-05 | MYL1 | 1.54 | T | A | -0.43372 | 0.1012 |
| rs62202078 | 2 | 211239367 | 2.02E-05 | MYL1 | 1.54 | G | A | -0.43372 | 0.1012 |
| rs62202080 | 2 | 211239848 | 2.02E-05 | MYL1 | 1.54 | A | G | -0.43372 | 0.1012 |
| rs62202081 | 2 | 211239981 | 2.02E-05 | MYL1 | 1.54 | C | A | -0.43372 | 0.1012 |
| rs1714631 | 6 | 107304447 | 2.09E-05 | C6orf203 | 1.58 | T | C | -0.45607 | 0.10661 |
| rs7960065 | 12 | 66905028 | 2.23E-05 | GRIP1 | 1.52 | G | T | 0.417776 | 0.097995 |
| rs7977592 | 12 | 66906325 | 2.29E-05 | GRIP1 | 1.51 | G | A | 0.411549 | 0.096684 |
| rs7977856 | 12 | 66906586 | 2.29E-05 | GRIP1 | 1.51 | T | A | 0.411549 | 0.096684 |
| rs12099510 | 12 | 66907635 | 2.29E-05 | GRIP1 | 1.51 | C | A | 0.411549 | 0.096684 |
| rs7965031 | 12 | 66908633 | 2.29E-05 | GRIP1 | 1.51 | A | G | 0.411549 | 0.096684 |
| rs12579414 | 12 | 66909059 | 2.29E-05 | GRIP1 | 1.51 | G | A | 0.411549 | 0.096684 |
| rs6581702 | 12 | 66910566 | 2.29E-05 | GRIP1 | 1.51 | G | A | 0.411549 | 0.096684 |
| rs7976246 | 12 | 66911142 | 2.38E-05 | GRIP1 | 1.51 | A | C | 0.411648 | 0.0969 |
| rs35666435 | 12 | 66911385 | 2.38E-05 | GRIP1 | 1.51 | G | C | 0.411648 | 0.0969 |
| rs7313182 | 12 | 66899026 | 2.38E-05 | GRIP1 | 1.52 | T | C | 0.415646 | 0.09785 |
| rs7299018 | 12 | 66899238 | 2.38E-05 | GRIP1 | 1.52 | A | G | 0.415646 | 0.09785 |
| rs61925948 | 12 | 66899853 | 2.38E-05 | GRIP1 | 1.52 | G | T | 0.415646 | 0.09785 |
| rs1493488 | 12 | 66901409 | 2.38E-05 | GRIP1 | 1.52 | C | T | 0.415646 | 0.09785 |
| rs1493490 | 12 | 66901824 | 2.38E-05 | GRIP1 | 1.52 | A | T | 0.415646 | 0.09785 |
| rs17102578 | 12 | 66902466 | 2.38E-05 | GRIP1 | 1.52 | T | C | 0.415646 | 0.09785 |
| rs13321825 | 3 | 14640547 | 2.38E-05 | CCDC174 | 1.61 | T | C | 0.47795 | 0.112518 |
| rs11613276 | 12 | 66907158 | 2.43E-05 | GRIP1 | 1.51 | A | C | 0.410182 | 0.096658 |
| rs7523309 | 1 | 83268117 | 2.44E-05 | Nothing | 1.55 | G | A | -0.43691 | 0.102983 |
| rs4633862 | 2 | 211214464 | 2.56E-05 | MYL1 | 1.56 | T | C | -0.44493 | 0.10514 |
| rs7960316 | 12 | 66905218 | 2.65E-05 | GRIP1 | 1.51 | C | T | 0.413722 | 0.097963 |
| rs658977 | 18 | 53213887 | 2.75E-05 | TCF4 | 1.74 | A | G | 0.556221 | 0.131952 |
| rs56243809 | 12 | 66900129 | 2.94E-05 | GRIP1 | 1.51 | C | A | 0.412424 | 0.098211 |
| rs75854150 | 11 | 127174873 | 3.01E-05 | KIRREL3 | 1.72 | C | T | -0.54246 | 0.129343 |
| rs1478620 | 11 | 127175854 | 3.01E-05 | KIRREL3 | 1.72 | A | G | -0.54246 | 0.129343 |
| rs4673517 | 2 | 211240288 | 3.05E-05 | MYL1 | 1.53 | C | T | -0.42466 | 0.101332 |
| rs11176239 | 12 | 66907528 | 3.14E-05 | GRIP1 | 1.51 | G | A | 0.40938 | 0.09784 |
| rs4772129 | 13 | 99392375 | 3.16E-05 | SLC15A1 | 2.14 | C | A | -0.76029 | 0.181764 |
| rs28513666 | 12 | 66905678 | 3.19E-05 | GRIP1 | 1.51 | A | C | 0.409054 | 0.097843 |
| rs11126894 | 2 | 83436237 | 3.21E-05 | Nothing | 1.61 | G | T | 0.476846 | 0.1141 |
| rs4285075 | 4 | 82645452 | 3.23E-05 | RASGEF1B | 1.58 | A | G | 0.459913 | 0.110078 |
| rs7138741 | 12 | 66913729 | 3.29E-05 | GRIP1 | 1.51 | A | C | 0.409237 | 0.098061 |
| rs1020964 | 11 | 127170177 | 3.30E-05 | KIRREL3 | 1.71 | A | C | -0.53791 | 0.128906 |
| rs1329914 | 9 | 17963940 | 3.34E-05 | SH3GL2 | 1.72 | G | A | -0.53974 | 0.129441 |
| rs16844359 | 2 | 211241745 | 3.36E-05 | MYL1 | 1.52 | C | T | -0.42171 | 0.10116 |
| rs1379836 | 2 | 211237918 | 3.38E-05 | MYL1 | 1.52 | G | A | -0.41707 | 0.100076 |
| rs2120750 | 15 | 26147045 | 3.42E-05 | LOC100128714 | 1.61 | T | G | -0.47528 | 0.114133 |
| rs7313809 | 12 | 66899381 | 3.45E-05 | GRIP1 | 1.50 | T | G | 0.407276 | 0.097837 |
| rs627685 | 18 | 53186092 | 3.46E-05 | TCF4 | 1.58 | C | T | 0.45764 | 0.109957 |
| rs17314018 | 2 | 83392185 | 3.57E-05 | Nothing | 1.61 | C | T | 0.475987 | 0.114566 |
| rs2012959 | 11 | 127170383 | 3.59E-05 | KIRREL3 | 1.71 | A | G | -0.53623 | 0.129116 |
| rs62202076 | 2 | 211239244 | 3.70E-05 | MYL1 | 1.52 | T | C | -0.4199 | 0.101273 |
| rs4936568 | 11 | 120825575 | 3.76E-05 | GRIK4 | 1.49 | A | G | -0.39666 | 0.095757 |
| rs2618198 | 11 | 127166513 | 3.78E-05 | KIRREL3 | 1.74 | G | A | -0.55359 | 0.133686 |
| rs5748748 | 22 | 17415572 | 3.86E-05 | GAB4 | 1.89 | C | T | 0.638286 | 0.154321 |
| rs11811473 | 1 | 230667238 | 3.90E-05 | PGBD5 | 1.50 | A | G | -0.40536 | 0.098058 |
| rs57122637 | 2 | 211231086 | 3.94E-05 | MYL1 | 1.52 | C | G | -0.42058 | 0.1018 |
| rs2614967 | 7 | 3627799 | 3.97E-05 | SDK1 | 1.54 | T | C | -0.43459 | 0.105237 |
| rs7488693 | 12 | 66906183 | 4.03E-05 | GRIP1 | 1.49 | G | A | 0.398819 | 0.096658 |
| rs7294455 | 12 | 66914386 | 4.20E-05 | GRIP1 | 1.49 | A | G | 0.398716 | 0.096855 |
| rs4144463 | 12 | 66914523 | 4.20E-05 | GRIP1 | 1.49 | T | C | 0.398716 | 0.096855 |
| rs1329913 | 9 | 17963948 | 4.34E-05 | SH3GL2 | 1.70 | A | G | -0.53085 | 0.129194 |
| rs4246194 | 10 | 128192049 | 4.44E-05 | C10orf90 | 1.62 | A | G | 0.484453 | 0.118054 |
| rs56920009 | 2 | 211211684 | 4.55E-05 | MYL1 | 1.53 | T | C | -0.42749 | 0.104318 |
| rs142003553 | 2 | 211213660 | 4.55E-05 | MYL1 | 1.53 | T | C | -0.42749 | 0.104318 |
| rs4632267 | 2 | 211214598 | 4.55E-05 | MYL1 | 1.53 | T | C | -0.42749 | 0.104318 |
| rs138457983 | 12 | 66916496 | 4.60E-05 | GRIP1 | 1.49 | T | C | 0.396769 | 0.096886 |
| rs2806905 | 13 | 46486234 | 4.65E-05 | ZC3H13 | 1.50 | G | A | 0.403964 | 0.098702 |
| rs146879561 | 12 | 66919850 | 4.72E-05 | GRIP1 | 1.49 | C | T | 0.398895 | 0.097552 |
| rs16916504 | 10 | 63452956 | 4.79E-05 | C10orf107 | 1.58 | G | A | 0.455391 | 0.111459 |
| rs6919878 | 6 | 1064872 | 4.86E-05 | LOC285768 | 1.55 | G | A | 0.441216 | 0.108083 |
| rs4144465 | 12 | 66914192 | 4.87E-05 | GRIP1 | 1.49 | A | G | 0.397299 | 0.097341 |
| rs9636107 | 18 | 53200117 | 4.88E-05 | TCF4 | 1.51 | A | G | 0.412436 | 0.101052 |
| rs6562920 | 13 | 76185399 | 4.92E-05 | UCHL3 | 1.48 | A | T | -0.39406 | 0.096598 |
| rs143532626 | 2 | 211214282 | 4.94E-05 | MYL1 | 1.54 | A | G | -0.42954 | 0.105324 |
| rs4577226 | 2 | 211214494 | 4.94E-05 | MYL1 | 1.54 | G | C | -0.42954 | 0.105324 |

†Beta (effect size) in this case is the unit of standard deviation explained by each SNP.

**Table S3: Changes in gene expression (microarray) correlated with EBV genome load**

| **Array tag** | **Gene** | **Chr** | **Beta†** | **SE** | **P value** |
| --- | --- | --- | --- | --- | --- |
| ILMN_1672278 | CXCL16 | 17 | 0.113706 | 0.018609 | 2.40E-09 |
| ILMN_1680343 | AGL | 1 | 0.039183 | 0.007199 | 9.25E-08 |
| ILMN_1749493 | ADARB2 | 10 | -0.0422 | 0.008793 | 2.27E-06 |
| ILMN_1777397 | MSX1 | 4 | -0.13284 | 0.028089 | 3.15E-06 |
| ILMN_1698186 | IFNA2 | 9 | -0.09781 | 0.020937 | 4.11E-06 |
| ILMN_1698179 | TAGLN3 | 3 | -0.03819 | 0.008225 | 4.68E-06 |
| ILMN_1704335 | FXR1 | 3 | 0.022558 | 0.004936 | 6.54E-06 |
| ILMN_1768783 | ATP11A | 13 | -0.01119 | 0.002474 | 8.07E-06 |
| ILMN_1787567 | TSC22D1 | 13 | -0.07823 | 0.017783 | 1.40E-05 |
| ILMN_1746515 | DNTT | 10 | -0.02517 | 0.005869 | 2.26E-05 |
| ILMN_1780132 | PELI2 | 14 | -0.03122 | 0.007279 | 2.26E-05 |
| ILMN_1703787 | ATP4B | 13 | 0.026846 | 0.006299 | 2.54E-05 |
| ILMN_1806607 | SFN | 1 | 0.035761 | 0.008423 | 2.72E-05 |
| ILMN_1692177 | TSC22D1 | 13 | -0.05643 | 0.013306 | 2.78E-05 |
| ILMN_1656837 | RBP1 | 3 | -0.06119 | 0.01458 | 3.35E-05 |
| ILMN_1670672 | TMEM37 | 2 | -0.03642 | 0.008711 | 3.59E-05 |
| ILMN_1663753 | PILRA | 7 | -0.07404 | 0.017853 | 4.13E-05 |
| ILMN_1759933 | MS4A6E | 11 | 0.063279 | 0.015331 | 4.48E-05 |
| ILMN_1812517 | FGF8 | 10 | -0.01845 | 0.0045 | 5.03E-05 |
| ILMN_1801616 | EMP1 | 12 | 0.039923 | 0.010164 | 0.000101 |
| ILMN_1675677 | TMPRSS3 | 21 | -0.10897 | 0.027916 | 0.000112 |
| ILMN_1751396 | BAG5 | 14 | 0.017634 | 0.004518 | 0.000112 |
| ILMN_1741768 | TMPRSS3 | 21 | -0.13382 | 0.034455 | 0.000121 |
| ILMN_1785732 | TNFAIP6 | 2 | -0.06838 | 0.017642 | 0.000125 |
| ILMN_1700024 | UST | 6 | -0.10172 | 0.026322 | 0.00013 |
| ILMN_1678493 | CHN1 | 2 | 0.01646 | 0.004261 | 0.000131 |
| ILMN_1808590 | GUCY1A3 | 4 | -0.09414 | 0.024577 | 0.000149 |
| ILMN_1788203 | HEY1 | 8 | -0.10129 | 0.026515 | 0.000155 |
| ILMN_1710075 | FAM89A | 1 | -0.06912 | 0.018216 | 0.000171 |
| ILMN_1701613 | RARRES3 | 11 | -0.05164 | 0.013639 | 0.000177 |
| ILMN_1692739 | ISLR2 | 15 | 0.016983 | 0.004494 | 0.000182 |
| ILMN_1771385 | GBP4 | 1 | -0.09562 | 0.025316 | 0.000183 |
| ILMN_1789961 | RETSAT | 2 | 0.039601 | 0.010512 | 0.000191 |
| ILMN_1653340 | RECQL5 | 17 | 0.012247 | 0.003256 | 0.000195 |
| ILMN_1655236 | GFPT2 | 5 | 0.032536 | 0.008663 | 0.000199 |
| ILMN_1758049 | NFIA | 1 | -0.01067 | 0.002854 | 0.000213 |
| ILMN_1740213 | ELOVL7 | 5 | -0.02115 | 0.005668 | 0.000218 |
| ILMN_1679417 | UPF2 | 10 | 0.020625 | 0.005545 | 0.000228 |
| ILMN_1674402 | TMEM71 | 8 | -0.02888 | 0.007853 | 0.000269 |
| ILMN_1727332 | ATPIF1 | 1 | 0.033129 | 0.009041 | 0.000282 |
| ILMN_1663569 | FTCD | 21 | 0.014073 | 0.003858 | 0.0003 |
| ILMN_1690484 | KIAA0895 | 7 | 0.012477 | 0.003475 | 0.000372 |
| ILMN_1788874 | SERPINA3 | 14 | 0.009871 | 0.002754 | 0.00038 |
| ILMN_1793101 | PAX4 | 7 | 0.008634 | 0.002409 | 0.000381 |
| ILMN_1802205 | RHOB | 2 | -0.0766 | 0.021374 | 0.000382 |
| ILMN_1675453 | HHIP | 4 | -0.01488 | 0.004168 | 0.000403 |
| ILMN_1730201 | DTNA | 18 | -0.04685 | 0.013155 | 0.000414 |
| ILMN_1729915 | PILRA | 7 | -0.03863 | 0.010875 | 0.000429 |
| ILMN_1800540 | CD55 | 1 | -0.05768 | 0.016303 | 0.000452 |
| ILMN_1735471 | ACHE | 7 | -0.00994 | 0.002818 | 0.000472 |
| ILMN_1782567 | GUCY1B3 | 4 | -0.06091 | 0.017279 | 0.000473 |
| ILMN_1736527 | TFCP2L1 | 2 | 0.01997 | 0.005666 | 0.000474 |
| ILMN_1743373 | DLL1 | 6 | -0.05251 | 0.014913 | 0.00048 |
| ILMN_1807042 | MARCKS | 6 | -0.03919 | 0.011136 | 0.000484 |
| ILMN_1699160 | ITK | 5 | 0.032461 | 0.009255 | 0.000505 |
| ILMN_1785570 | SUSD3 | 9 | -0.0487 | 0.013951 | 0.000537 |
| ILMN_1717163 | F13A1 | 6 | -0.10259 | 0.029525 | 0.000569 |
| ILMN_1710644 | MARVELD3 | 16 | -0.0099 | 0.002857 | 0.000588 |
| ILMN_1657058 | PPIL6 | 6 | -0.00955 | 0.002758 | 0.00059 |
| ILMN_1758281 | CALCRL | 2 | -0.02374 | 0.006855 | 0.000592 |
| ILMN_1670087 | FBXO43 | 8 | 0.015505 | 0.004478 | 0.000594 |
| ILMN_1690646 | SATB1 | 3 | -0.0113 | 0.003276 | 0.000625 |
| ILMN_1743397 | PIGW | 17 | 0.051603 | 0.015037 | 0.000664 |
| ILMN_1743397 | PIGW | 17 | 0.051603 | 0.015037 | 0.000664 |
| ILMN_1663453 | HIST1H2AI | 6 | 0.01861 | 0.005436 | 0.000684 |
| ILMN_1716983 | LILRA2 | 19 | -0.06382 | 0.018754 | 0.000735 |
| ILMN_1716983 | LILRA2 | 19 | -0.06382 | 0.018754 | 0.000735 |
| ILMN_1716983 | LILRA2 | 19 | -0.06382 | 0.018754 | 0.000735 |
| ILMN_1716983 | LILRA2 | 19 | -0.06382 | 0.018754 | 0.000735 |
| ILMN_1779015 | ZNF467 | 7 | -0.01953 | 0.00576 | 0.000769 |
| ILMN_1724253 | NHLH1 | 1 | 0.032062 | 0.009459 | 0.000771 |
| ILMN_1806754 | GLDC | 9 | 0.064651 | 0.019109 | 0.000788 |
| ILMN_1743032 | CTSS | 1 | -0.03484 | 0.010341 | 0.000829 |
| ILMN_1702534 | CD244 | 1 | -0.05817 | 0.017288 | 0.000841 |
| ILMN_1703776 | CNTNAP5 | 2 | -0.00881 | 0.00262 | 0.000852 |
| ILMN_1796146 | EIF4E3 | 3 | -0.02479 | 0.007382 | 0.000859 |
| ILMN_1707312 | NFIL3 | 9 | -0.05916 | 0.017687 | 0.000903 |
| ILMN_1707077 | SORT1 | 1 | 0.050211 | 0.015027 | 0.000914 |
| ILMN_1715332 | TTC21A | 3 | -0.03202 | 0.009593 | 0.000924 |
| ILMN_1740024 | NAALAD2 | 11 | -0.02598 | 0.007813 | 0.000966 |
| ILMN_1779966 | PAIP2 | 5 | 0.011652 | 0.003506 | 0.000974 |

†Beta (effect size) expresses the relationship between EBV load and level of gene expression; betas greater than 0 represent a positive correlation, while betas smaller than 0 represent a negative correlation.
